# The genome sequence of *Aplidium turbinatum *(Savigny 1816), a colonial sea squirt

**DOI:** 10.12688/wellcomeopenres.17785.1

**Published:** 2022-03-22

**Authors:** John Bishop, Joanna Harley, Robert Mrowicki, Marine Biological Association Genome Acquisition Lab, Darwin Tree of Life Barcoding collective, Wellcome Sanger Institute Tree of Life programme, Wellcome Sanger Institute Scientific Operations: DNA Pipelines collective, Tree of Life Core Informatics collective, Darwin Tree of Life Consortium

**Affiliations:** 1 Marine Biological Association, Plymouth, Devon, UK; 2 Natural History Museum, London, UK

**Keywords:** Aplidium turbinatum, sea squirt, genome sequence, chromosomal, Ascidiacea

## Abstract

We present a genome assembly from an individual *Aplidium turbinatum *(Chordata; Ascidiacea; Aplousobranchia; Polyclinidae). The genome sequence is 605 megabases in span. The majority of the assembly (99.98%) is scaffolded into 18 chromosomal pseudomolecules. The complete mitochondrial genome was also assembled and is 18.4 kilobases in length.

## Species taxonomy

Eukaryota; Metazoa; Chordata; Tunicata; Ascidiacea; Aplousobranchia; Polyclinidae; Aplidium; *Aplidium turbinatum* (Savigny 1816) (NCBI:txid2771288).

## Background

The polyclinid ascidian *Aplidium turbinatum* (formerly known as *Sidnyum turbinatum –* see ( Monniot & Monniot, 1987)) has a European distribution from Norway to the Mediterranean. It is frequently encountered in shallow water around the coasts of Great Britain and Ireland.

Colonies comprise a number of lobes or ‘heads’ with flat tops, tapering towards their common attached base. Up to 12 (rarely 25) zooids approximately 5 mm long are embedded vertically in each colony lobe, with their separate eight-lobed inhalant openings in the flat upper surface, arranged around a single exhalant opening. The colonial tunic in which the zooids are embedded is unusually thin for a polyclinid and transparent, meaning that the zooids inside can be seen clearly. Pigmentation in a variety of possible colours picks out the endostyle and the structure of the branchial basket of the zooids, while the inhalant openings are often also pigmented.

A cytotoxic substance, turbinamide, has been isolated from *A. turbinatum* ( Aiello *et al.*, 2001). The cytotoxic effect was found to be selective, acting against neuronal cells but not immune-system cells.

## Genome sequence report

**Figure 1.**
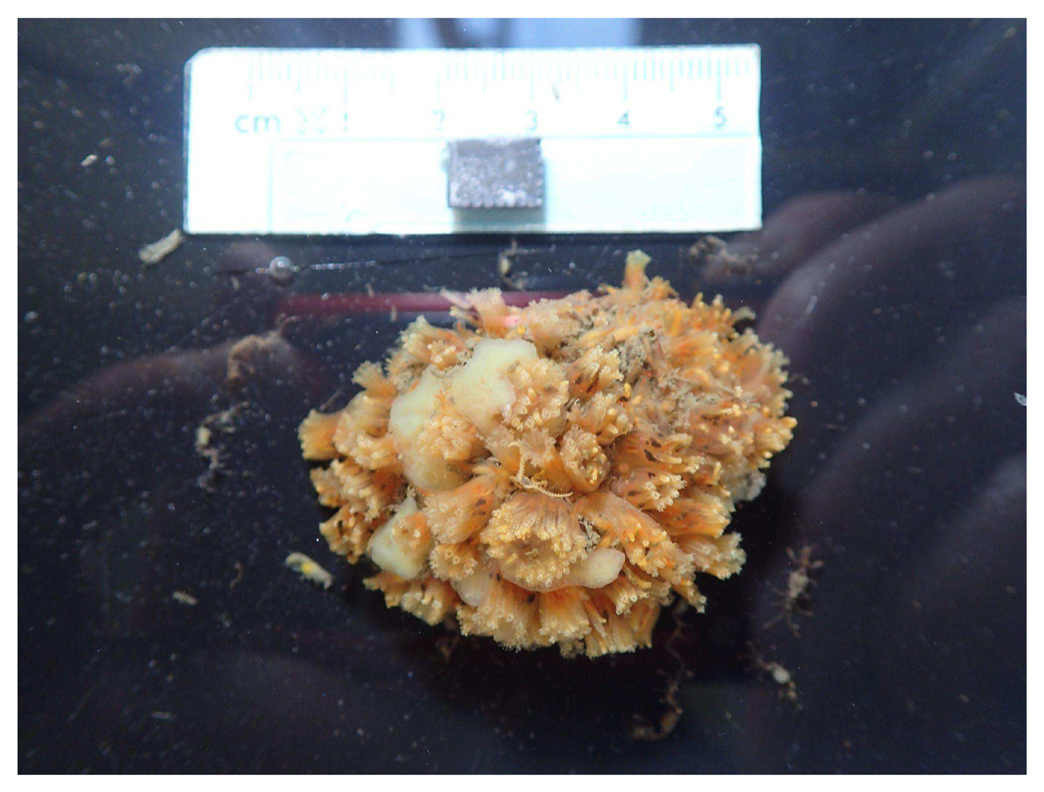
Image of the *Aplidium turbinatum* specimen taken during preservation and processing.

The genome was sequenced from a single monoecious hermaphrodite *A. turbinatum* clonal colony ( Figure 1) collected from Queen Anne's Battery Marina visitors' pontoon, Plymouth, UK. A total of 65-fold coverage in Pacific Biosciences single-molecule HiFi long reads and 85-fold coverage in 10X Genomics read clouds were generated. Primary assembly contigs were scaffolded with chromosome conformation Hi-C data. Manual assembly curation corrected 37 missing/misjoins and removed 10 haplotypic duplications, reducing the assembly size by 0.54%, the scaffold number by 43.48%, and the scaffold N50 by 0.71%.

**Figure 2.**
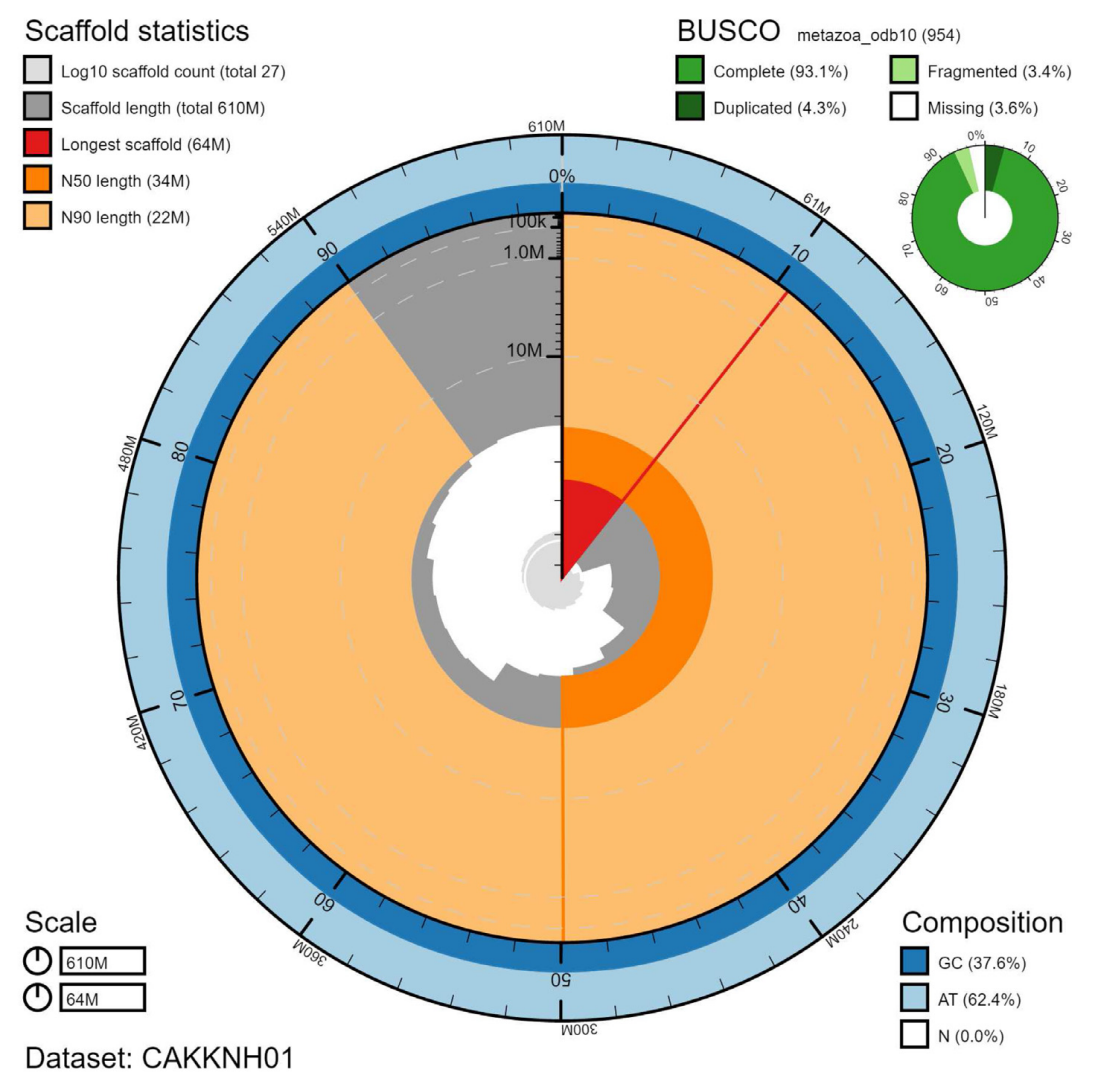
Genome assembly of *Aplidium turbinatum*, kaAplTurb1.1: metrics. The BlobToolKit Snailplot shows N50 metrics and BUSCO gene completeness. The main plot is divided into 1,000 size-ordered bins around the circumference with each bin representing 0.1% of the 605,507,883 bp assembly. The distribution of scaffold lengths is shown in dark grey with the plot radius scaled to the longest scaffold present in the assembly (64,349,347 bp, shown in red). Orange and pale-orange arcs show the N50 and N90 scaffold lengths (34,009,763 and 22,221,527 bp), respectively. The pale grey spiral shows the cumulative scaffold count on a log scale with white scale lines showing successive orders of magnitude. The blue and pale-blue area around the outside of the plot shows the distribution of GC, AT and N percentages in the same bins as the inner plot. A summary of complete, fragmented, duplicated and missing BUSCO genes in the metazoa_odb10 set is shown in the top right. An interactive version of this figure is available at https://blobtoolkit.genomehubs.org/view/kaAplTurb1.1/dataset/CAKKNH01/snail.

The final assembly has a total length of 605 Mb in 26 sequence scaffolds with a scaffold N50 of 34.0 Mb ( Table 1). The majority, 99.88%, of the assembly sequence was assigned to 11 chromosomal-level scaffolds, representing 18 autosomes (numbered by sequence length) ( Figure 2– Figure 5; Table 2). The assembly was curated to 18 chromosomes and oriented with the centromere on the left. Of note, telomere regions on chromosomes are all at half coverage. A few small scaffolds remain unlocalised due to lack of Hi-C signal.

**Table 1.** Genome data for *Aplidium turbinatum*, kaAplTurb1.1.

*Project accession data*	
Assembly identifier	kaAplTurb1.1
Species	*Diadumene lineata*
Specimen	kaAplTurb1
NCBI taxonomy ID	2771288
BioProject	PRJEB45189
BioSample ID	SAMEA7536566
Isolate information	kaAplTurb1, monoecious hermaphrodite clonal colony
*Raw data accessions*	
PacificBiosciences SEQUEL II	ERR6412042, ERR6412043
10X Genomics Illumina	ERR6286724-ERR6286727
Hi-C Illumina	ERR6286728
PolyA RNA-Seq Illumina	ERR6745738, ERR6745739
*Genome assembly*	
Assembly accession	GCA_918807975.1
*Accession of alternate haplotype*	GCA_918843895.1
Span (Mb)	605
Number of contigs	79
Contig N50 length (Mb)	14.9
Number of scaffolds	26
Scaffold N50 length (Mb)	34.0
Longest scaffold (Mb)	64.3
BUSCO * genome score	C:93.1%[S:88.8%,D:4.3%],F:3.4 %,M:3.6%,n:954

*BUSCO scores based on the metazoa_odb10 BUSCO set using v5.1.2. C= complete [S= single copy, D=duplicated], F=fragmented, M=missing, n=number of orthologues in comparison. A full set of BUSCO scores is available at https://blobtoolkit.genomehubs.org/view/kaAplTurb1.1/dataset/CAKKNH01/busco.

**Figure 3.**
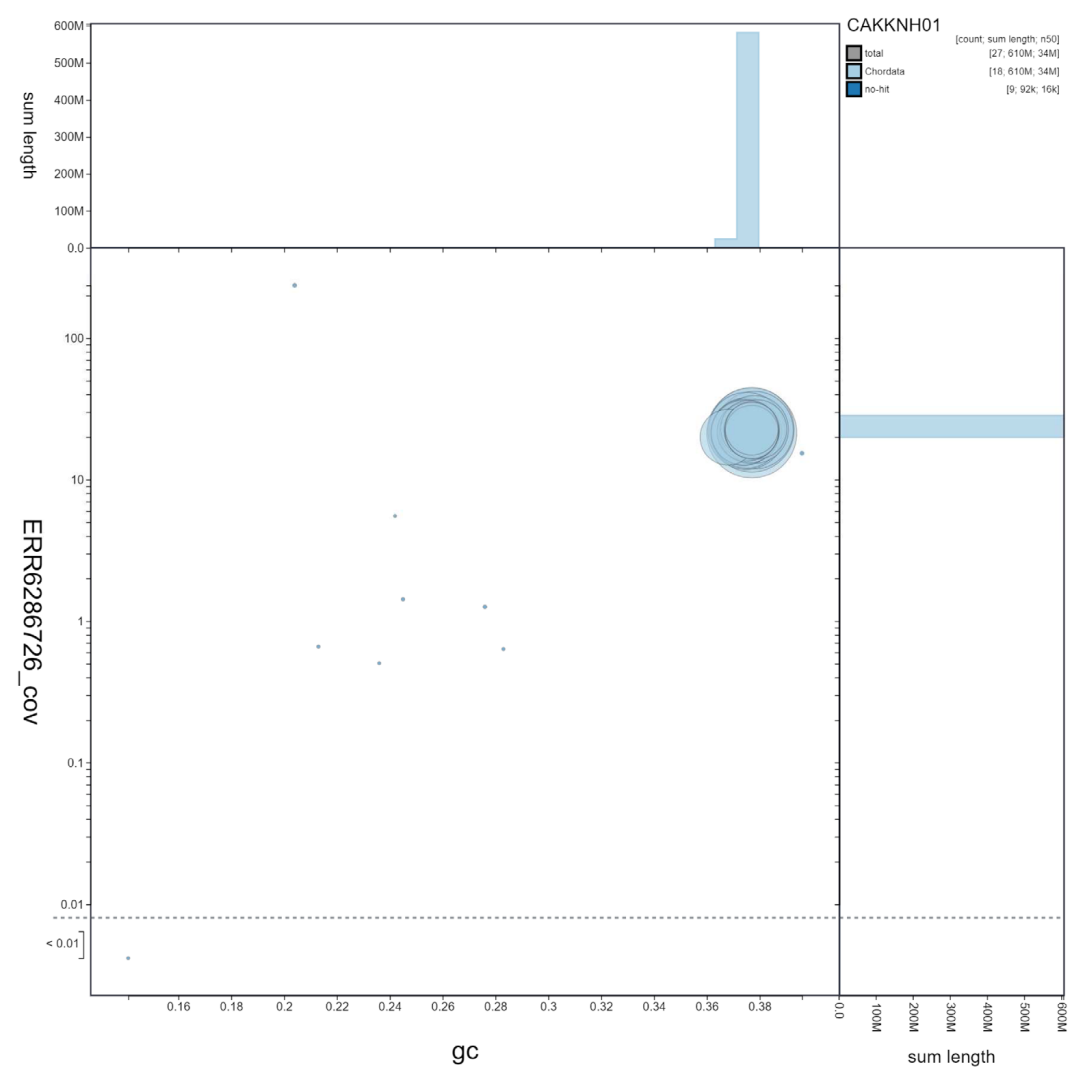
Genome assembly of *Aplidium turbinatum*, kaAplTurb1.1: GC coverage. BlobToolKit GC-coverage plot. Scaffolds are coloured by phylum. Circles are sized in proportion to scaffold length. Histograms show the distribution of scaffold length sum along each axis. An interactive version of this figure is available at https://blobtoolkit.genomehubs.org/view/kaAplTurb1.1/dataset/CAKKNH01/blob.

**Figure 4.**
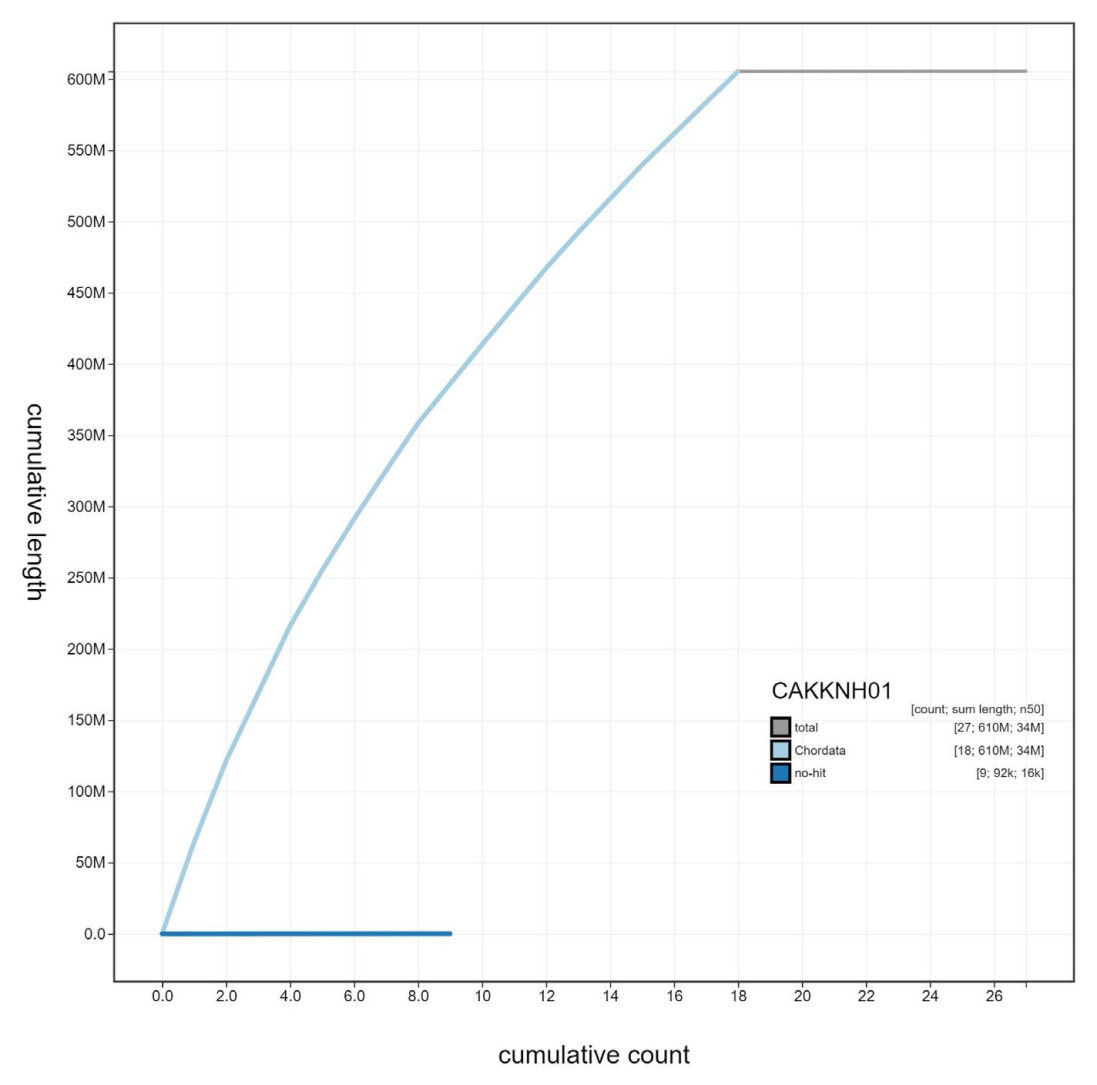
Genome assembly of *Aplidium turbinatum*, kaAplTurb1.1: cumulative sequence. BlobToolKit cumulative sequence plot. The grey line shows cumulative length for all scaffolds. Coloured lines show cumulative lengths of scaffolds assigned to each phylum using the buscogenes taxrule. An interactive version of this figure is available at https://blobtoolkit.genomehubs.org/view/kaAplTurb1.1/dataset/CAKKNH01/cumulative.

**Figure 5.**
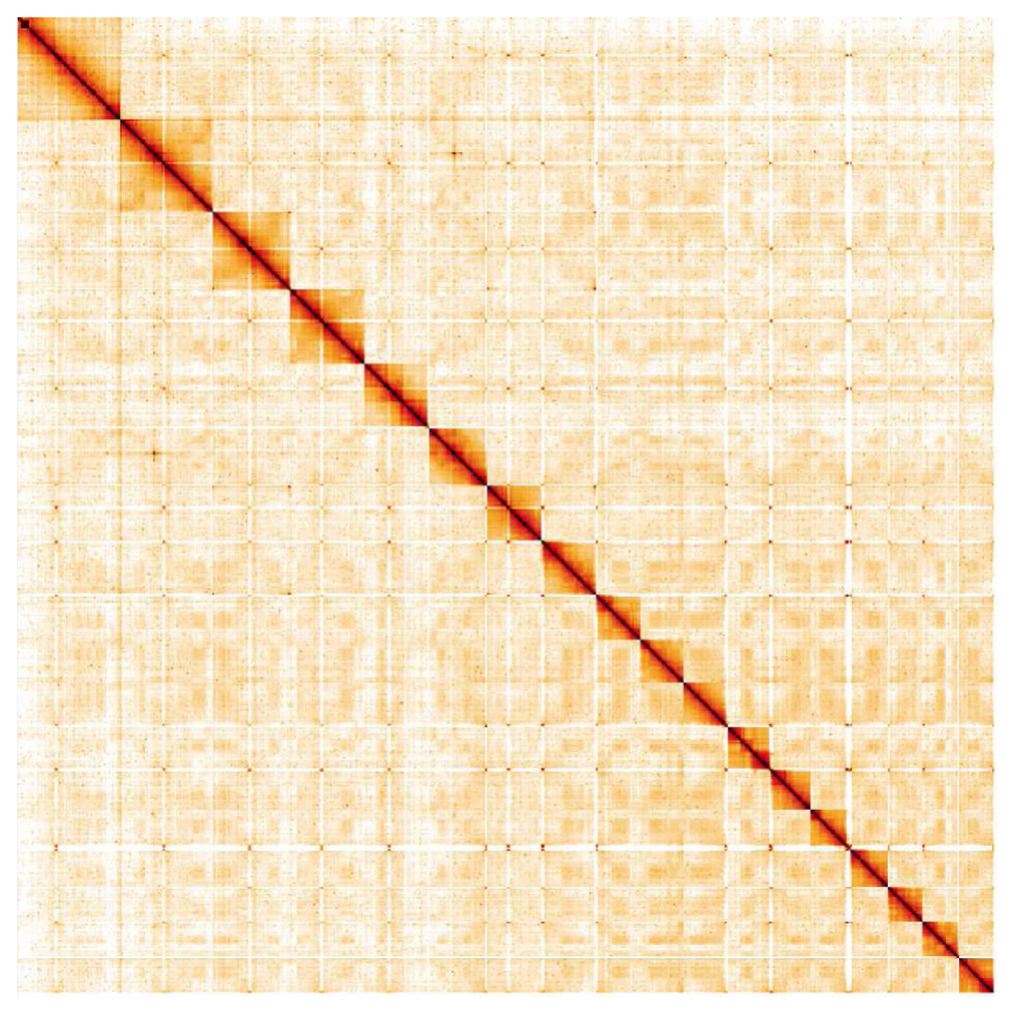
Genome assembly of *Aplidium turbinatum*, kaAplTurb1.1: Hi-C contact map. Hi-C contact map of the kaAplTurb1.1 assembly, visualised in HiGlass. Chromosomes are arranged in size order from left to right and top to bottom. The interactive Hi-C map can be viewed here.

**Table 2.** Chromosomal pseudomolecules in the genome assembly of *Aplidium turbinatum*, kaAplTurb1.1.

INSDC accession	Chromosome	Size (Mb)	GC%
OU974069.1	1	42.01	35.3
OU974070.1	2	33.73	35.3
OU974071.1	3	23.14	35.6
OU974072.1	4	19.48	35.2
OU974073.1	5	18.71	35.4
OU974074.1	6	18.65	35.3
OU974075.1	7	17.67	35.4
OU974076.1	8	15.82	35.3
OU974077.1	9	15.30	35.3
OU974078.1	10	14.93	35.3
OU974079.1	11	14.14	35.2
OU974080.1	12	14.10	35.2
OU974083.1	13	13.13	35.1
OU974081.1	14	13.25	35.3
OU974082.1	15	13.16	35.4
OU974084.1	16	12.63	35.0
OU974085.1	MT	0.02	37.4
-	Unplaced	13.14	33.4

The assembly has a BUSCO v5.1.2 ( Manni *et al.*, 2021) completeness of 93.1% (single 88.8%, duplicated 4.3%) using the metazoa_odb10 reference set (n=954). While not fully phased, the assembly deposited is of one haplotype. Contigs corresponding to the second haplotype have also been deposited.

## Methods

### Sample acquisition and DNA extraction

A single monoecious hermaphrodite *A. turbinatum* clonal colony (kaAplTurb1) was collected by hand from Queen Anne's Battery Marina visitors' pontoon, Plymouth, UK (latitude 50.3644, longitude -4.1320) by John Bishop, Joanna Harley (both Marine Biological Association) and Rob Mrowicki (Natural History Museum). The specimen was identified by John Bishop and snap-frozen in liquid nitrogen.

DNA was extracted at the Tree of Life laboratory, Wellcome Sanger Institute. The kaAplTurb1 sample was weighed and dissected on dry ice with tissue set aside for Hi-C and RNA sequencing. Tissue was disrupted using a Nippi Powermasher fitted with a BioMasher pestle. Fragment size analysis of 0.01–0.5 ng of DNA was then performed using an Agilent FemtoPulse. High molecular weight (HMW) DNA was extracted using the Qiagen MagAttract HMW DNA extraction kit. Low molecular weight DNA was removed from a 200-ng aliquot of extracted DNA using 0.8X AMpure XP purification kit prior to 10X Chromium sequencing; a minimum of 50 ng DNA was submitted for 10X sequencing. HMW DNA was sheared into an average fragment size between 12–20 kb in a Megaruptor 3 system with speed setting 30. Sheared DNA was purified by solid-phase reversible immobilisation using AMPure PB beads with a 1.8X ratio of beads to sample to remove the shorter fragments and concentrate the DNA sample. The concentration of the sheared and purified DNA was assessed using a Nanodrop spectrophotometer and Qubit Fluorometer and Qubit dsDNA High Sensitivity Assay kit. Fragment size distribution was evaluated by running the sample on the FemtoPulse system.

RNA was extracted from kaAplTurb1 in the Tree of Life Laboratory at the WSI using TRIzol, according to the manufacturer’s instructions. RNA was then eluted in 50 μl RNAse-free water and its concentration RNA assessed using a Nanodrop spectrophotometer and Qubit Fluorometer using the Qubit RNA Broad-Range (BR) Assay kit. Analysis of the integrity of the RNA was done using Agilent RNA 6000 Pico Kit and Eukaryotic Total RNA assay.

### Sequencing

Pacific Biosciences HiFi circular consensus and 10X Genomics Chromium read cloud sequencing libraries were constructed according to the manufacturers’ instructions. Sequencing was performed by the Scientific Operations core at the Wellcome Sanger Institute on Pacific Biosciences SEQUEL II (HiFi), Illumina NovaSeq 6000 (10X) and Illumina HiSeq 4000 (RNA-Seq) instruments. Hi-C data were generated in the Tree of Life laboratory from remaining tissue of kaAplTurb1 using the Arima v2 kit and sequenced on a NovaSeq 6000 instrument.

### Genome assembly

Assembly was carried out with Hifiasm ( Cheng *et al.*, 2021); haplotypic duplication was identified and removed with purge_dups ( Guan *et al.*, 2020). One round of polishing was performed by aligning 10X Genomics read data to the assembly with longranger align, calling variants with freebayes ( Garrison & Marth, 2012). The assembly was then scaffolded with Hi-C data ( Rao *et al.*, 2014) using SALSA2 ( Ghurye *et al.*, 2019). The assembly was checked for contamination as described previously ( Howe *et al.*, 2021). Manual curation was performed using HiGlass ( Kerpedjiev *et al.*, 2018) and Pretext. The mitochondrial genome was assembled using MitoHiFi ( Uliano-Silva *et al.*, 2021), which performs annotation using MitoFinder ( Allio *et al.*, 2020). The genome was analysed and BUSCO scores generated within the BlobToolKit environment ( Challis *et al.*, 2020). Table 3 contains a list of all software tool versions used, where appropriate.

**Table 3.** Software tools used.

Software tool	Version	Source
Hifiasm	0.14-r312	Cheng *et al.*, 2021
purge_dups	1.2.3	Guan *et al.*, 2020
SALSA2	2.2	Ghurye *et al.*, 2019
longranger align	2.2.2	https://support.10xgenomics. com/genome-exome/software/ pipelines/latest/advanced/other- pipelines
freebayes	1.3.1-17- gaa2ace8	Garrison & Marth, 2012
MitoHiFi	2.0	Uliano-Silva *et al.*, 2021
HiGlass	1.11.6	Kerpedjiev *et al.*, 2018
PretextView	0.2.x	https://github.com/wtsi-hpag/ PretextView
BlobToolKit	3.0.5	Challis *et al.*, 2020

### Ethics/compliance issues

The materials that have contributed to this genome note have been supplied by a Darwin Tree of Life Partner. The submission of materials by a Darwin Tree of Life Partner is subject to the Darwin Tree of Life Project Sampling Code of Practice. By agreeing with and signing up to the Sampling Code of Practice, the Darwin Tree of Life Partner agrees they will meet the legal and ethical requirements and standards set out within this document in respect of all samples acquired for, and supplied to, the Darwin Tree of Life Project. Each transfer of samples is further undertaken according to a Research Collaboration Agreement or Material Transfer Agreement entered into by the Darwin Tree of Life Partner, Genome Research Limited (operating as the Wellcome Sanger Institute), and in some circumstances other Darwin Tree of Life collaborators.

## Data availability

European Nucleotide Archive: Aplidium turbinatum (a colonial sea squirt). Accession number PRJEB45189; https://identifiers.org/ena.embl/PRJEB45189.

The genome sequence is released openly for reuse. The *A. turbinatum* genome sequencing initiative is part of the Darwin Tree of Life (DToL) project. All raw sequence data and the assembly have been deposited in INSDC databases. The genome will be annotated using the RNA-Seq data and presented through the Ensembl pipeline at the European Bioinformatics Institute. Raw data and assembly accession identifiers are reported in Table 1.

## Author information

Members of the Marine Biological Association Genome Acquisition Lab are listed here: https://doi.org./10.5281/zenodo.5913830.

Members of the Darwin Tree of Life Barcoding collective are listed here: https://doi.org/10.5281/zenodo.5744972.

Members of the Wellcome Sanger Institute Tree of Life programme are listed here: https://doi.org/10.5281/zenodo.6125027.

Members of Wellcome Sanger Institute Scientific Operations: DNA Pipelines collective are listed here: https://doi.org/10.5281/zenodo.5746904.

Members of the Tree of Life Core Informatics collective are listed here: https://doi.org/10.5281/zenodo.6125046.

Members of the Darwin Tree of Life Consortium are listed here: https://doi.org/10.5281/zenodo.5638618.
