# Supplementary material for: The genome sequence of Aplidium turbinatum (Savigny 1816), a colonial sea squirt
Source: Wellcome Open Res. 2022 Mar 22;7:106. [Version 1] doi: 10.12688/wellcomeopenres.17785.1 (PMC9171297; doi:10.12688/wellcomeopenres.17785.1)
Supplement: Peer review history

# Peer review: Reviewer response for version 1

**DOI:** 10.21956/wellcomeopenres.19684.r50737

**Published:** 2022-06-06

**Authors:** Alan Le Moan

**Affiliations:** 1 Department of Marine Sciences, Tjärnö Marine Laboratory, University of Gothenburg, Strömstad, Sweden

I went through the manuscript by Bishop et al. presenting the reference genome of the tunicate *Aplidium turbinatum*. The manuscript is clear and present a high quality reference genome. Overall, I think the reference genome looks really good and that the article can be Approved for indexing.

 I have identified a couple of points that could be clarify in the text:

1. What was the purpose of sequencing 65-fold coverage in Pac Bio HiFi and 85-fold in 10X Genomic and not relying only on PacBio HiFi?
2. I think that the following sentence could benefits from some “The assembly was curated to 18 chromosomes and oriented with the centromere on the left.”:
  a) How was the curation performed? Manually with PretextView?
  b) Was the “real” number of chromosome known from previous studies? Was this information used for the curation?
3. Same with the following sentence: “Of note, telomere regions on chromosomes are all at half coverage”:
  a) How to explain these differences in coverage?
  b) Perhaps a link to a figure or a table would help here.

Are sufficient details of methods and materials provided to allow replication by others?

Yes

Is the rationale for creating the dataset(s) clearly described?

Yes

Are the datasets clearly presented in a useable and accessible format?

Yes

Are the protocols appropriate and is the work technically sound?

Yes

Reviewer Expertise:

Population genomics

I confirm that I have read this submission and believe that I have an appropriate level of expertise to confirm that it is of an acceptable scientific standard.

# Peer review: Reviewer response for version 1

**DOI:** 10.21956/wellcomeopenres.19684.r49385

**Published:** 2022-04-19

**Authors:** Richard Edwards

**Affiliations:** 1 School of Biotechnology and Biomolecular Sciences, University of New South Wales, Sydney, NSW, Australia

Sea squirts represent a taxonomically important group of invertebrate animals with some primitive vertebrate features including a notochord. It is therefore of great interest to increase the number of available high-quality sea squirt genomes. This manuscript reports the core assembly details for a chromosome-level assembly of *Aplidium turbinatum* (Savigny 1816), using high-coverage high-quality modern sequencing and assembly methods. The final chromosome-level genome assembly appears to be in very good shape. 

 Overall, I am convinced by the quality of the work and the final product. However, I have identified a few areas where additional information would be useful and/or there might be room for additional (low effort) improvement at the authors' discretion.

**MINOR COMMENTS**

1.  "Colonies comprise a number of lobes or ‘heads’ with flat tops, tapering towards their common attached base. Up to 12 (rarely 25) zooids, approximately 5 mm long are embedded vertically in each colony lobe, with their separate eight-lobed inhalant openings in the flat upper surface, arranged around a single exhalant opening."
  This description is a little unclear. Are there multiple inhalent openings, each of eight lobes? Approx. how many in total? "A number" is a bit too vague. "Up to 12 (rarely 25)" also does not make sense. 25 is greater than 12. Maybe give the typical number "(up to 25)"?
2. For comparison purposes, BUSCO analysis needs to specify whether Augustus or Metaeuk was used for the gene prediction, as it can change the results quite substantially.
3. Whilst it is implied, it is not explicitly stated whether the software used for genome assembly, scaffolding, and polishing were run with default settings.
4. Given the 65X coverage of HiFi data and its random error profile, versus the biased error profile of the 10x Genomics (Illumina) reads, I wonder whether more accurate polishing would have been achieved using the HiFi data alone? Was 10x data used primarily to repair indel errors? Or were single base variants also "corrected". Ideally, Merqury kmer analysis using the HiFi reads would be used to assess the ideal polishing strategy.
5. I would like to see a little more detail regarding the manual curation and correction of misassemblies. The method implies that this was done solely on the basis of the Hi-C contact maps. Is this right? It is clear that seven misjoins were corrected to generate the 18 autosomes from 11 scaffolds. What was the nature of the other 30 corrections? Were these all correcting "missing joins" to improve the chromosome scaffolding?
6. Was any effort made to gap-fill the scaffolded assembly once the false duplications were removed?
7. "Of note, telomere regions on chromosomes are all at half coverage." Please expand on this. What exactly is meant by "telomere region"? Is this more than just the telomeres? Why is it of note and what are the implications? (Are they very heterozygous and thus only covered by one haplotype's reads?) Are all the autosomes telomere-to-telomere, or are any telomeres missing?
8. Table 2 appears to be missing two autosomes (17 and 18).

Are sufficient details of methods and materials provided to allow replication by others?

Partly

Is the rationale for creating the dataset(s) clearly described?

Yes

Are the datasets clearly presented in a useable and accessible format?

Yes

Are the protocols appropriate and is the work technically sound?

Yes

Reviewer Expertise:

Genome assembly, genome curation, evolutionary genomics and bioinformatics.

I confirm that I have read this submission and believe that I have an appropriate level of expertise to confirm that it is of an acceptable scientific standard.
